# CD73 Predicts Favorable Prognosis in Patients with Nonmuscle-Invasive Urothelial Bladder Cancer

**DOI:** 10.1155/2015/785461

**Published:** 2015-10-12

**Authors:** Marian S. Wettstein, Lorenz Buser, Thomas Hermanns, Filip Roudnicky, Daniel Eberli, Philipp Baumeister, Tullio Sulser, Peter Wild, Cédric Poyet

**Affiliations:** ^1^Department of Urology, University Hospital, University of Zurich, 8091 Zurich, Switzerland; ^2^Institute of Surgical Pathology, University Hospital, University of Zurich, 8091 Zurich, Switzerland; ^3^Institute of Pharmaceutical Sciences, ETH Zurich, 8093 Zurich, Switzerland

## Abstract

*Aims*. CD73 is a membrane associated 5′-ectonucleotidase that has been proposed as prognostic biomarker in various solid tumors. The aim of this study is to evaluate CD73 expression in a cohort of patients with primary bladder cancer in regard to its association with clinicopathological features and disease course. *Methods*. Tissue samples from 174 patients with a primary urothelial carcinoma were immunohistochemically assessed on a tissue microarray. Associations between CD73 expression and retrospectively obtained clinicopathological data were evaluated by contingency analysis. Survival analysis was performed to investigate the predictive value of CD73 within the subgroup of pTa and pT1 tumors in regard to progression-free survival (PFS). *Results*. High CD73 expression was found in 46 (26.4%) patients and was significantly associated with lower stage, lower grade, less adjacent carcinoma in situ and with lower Ki-67 proliferation index. High CD73 immunoreactivity in the subgroup of pTa and pT1 tumors (*n* = 158) was significantly associated with longer PFS (HR: 0.228; *p* = 0.047) in univariable Cox regression analysis. *Conclusion*. High CD73 immunoreactivity was associated with favorable clinicopathological features. Furthermore, it predicts better outcome in the subgroup of pTa and pT1 tumors and may thus serve as additional tool for the selection of patients with favorable prognosis.

## 1. Introduction

Bladder cancer (BC)—the 11th most commonly diagnosed cancer worldwide [1]—presents in up to 80% of all patients either as a noninvasive papillary carcinoma (pTa) or as a carcinoma invading the submucosal connective tissue (pT1) [2, 3]. However, approximately 70% of these superficial tumors recur and up to a quarter even progress into muscle invasive bladder cancer (MIBC) [4]. Since close cystoscopic monitoring of recurrence and progression after initial transurethral resection of the tumor causes immense healthcare costs [5], accurate markers are needed in addition to clinicopathological factors [6] to individualize postoperative follow-up schedules [7–9]. Although numerous studies have evaluated the predictive value of different biomarkers in regard to progression of superficial bladder cancer [10–19], none of them has an established role in daily clinical practice.

CD73 (NT5E, ecto-5′-nucleotidase) is a glycosylphosphatidylinositol- (GPI-) anchored cell-surface enzyme that plays a crucial role in the purinergic signalling pathway by dephosphorylating AMP (adenosine monophosphate) into adenosine [20, 21]. Extracellular adenosine itself is involved in tumor immunoescape and invasion of tumor cells [22], while nonenzymatic functions of CD73 are related to cell adhesion and migration of tumor cells [23, 24].

CD73 expression has been investigated in many different cancer cell lines and human tumor biopsies so far and seems to play an important role in cancer development [20, 25, 26]. The role of CD73 in bladder BC is not well known and controversial [27–30]. Furthermore, neither larger expression studies of CD73 in BC biopsies nor studies investigating the predictive ability of this marker have been published so far. The aim of the present study is to evaluate the association between CD73 expression and tumor progression in a large cohort of patients with primary BC.

## 2. Material and Methods

Tissue microarrays (TMA) were constructed from 348 formalin-fixed, paraffin-embedded urothelial BC tissues from 174 patients as previously described [31]. Tumor samples were represented in duplicate tissue cores with a diameter of 1 mm. The collection of the specimens was performed by the Institute of Surgical Pathology, University Hospital, Zurich, Switzerland, between 1990 and 2006. The tissue samples in TMA represent a series of 174 consecutive (nonselected) primary urothelial bladder tumors consisting of 90 pTa, 68 pT1, and 16 ≥ pT2 tumors. A board-certified pathologist (Peter Wild) reevaluated the hematoxylin-and-eosin-stained slides of all specimens. Tumor stage and grade were assigned according to UICC and WHO criteria. Additionally, to analyse the immunoreactivity of CD73 in normal urothelium, eight slides were cut from formalin-fixed, paraffin-embedded urothelium of the bladder neck of patients without any history of urothelial dysplasia or BC.

Retrospective clinical follow-up data were available for all the 174 patients (100%). The median follow-up period for the entire cohort was 110.6 months ranging from 32.4 to 226.8 months. Unfortunately, a proper analysis of adjuvant bladder instillation therapy (BCG or chemotherapy) could not be performed due to missing data in about 50% of the patients. The TMA and its clinical cohort have been previously published [32]. Descriptive characteristics of the cohort are depicted in Table 1. The study was approved by the Cantonal Scientific Ethics Committee Zurich (http://www.kek.zh.ch, approval number: StV-NR. 25/2007).

TMA was freshly cut and used on 3 *μ*m paraffin sections as described previously [33]. The immunohistochemical detection of CD73 on tissue samples was performed by use of Anti-NT5E rabbit polyclonal antibody from Sigma Chemical Company, Saint Louis, United States (dilution 1 : 500). Clone MIB-1 (dilution 1 : 50; Dako, Glostrup, Denmark) was used for the detection of Ki-67. Immunohistochemical studies utilised an avidin-biotin peroxidase method with a diaminobenzidine (DAB) chromogen. After antigen retrieval (microwave oven for 30 min at 250 W), immunohistochemistry was conducted using a NEXES autostainer (Ventana, Tucson, AZ, USA) following the manufacturer's instructions.

Two experienced pathologists (Lorenz Buser and Peter Wild) evaluated all slides. Membranous CD73 immunoreactivity in the basal layer was assessed by using a semiquantitative three-scale scoring system ranging from 0 to 2+ (score 0: no staining; score 1+: weak staining; score 2+: strong staining).

In the situation of observing different staining intensities between the duplicate tissue cores, the core with more representative tumor tissue was chosen. If both duplicate tissue cores with different staining intensities demonstrate a comparable amount of representative tumor tissue, the intensity of the core with more homogenous staining intensity was selected. Immunoreactivity of CD73 was dichotomized for analytical purposes into a CD73 low-expression group (containing scores 0 and 1+) and a CD73 high-expression group (containing score 2+). The percentage of Ki-67 positive cells of each specimen was determined as described previously [34]. More than 10% of positive tumor cells were defined as a high Ki-67 labelling index [35].

Statistical analyses were performed with SPSS version 22 (IBM, Armonk, USA). *p* values < 0.05 were considered as statistically significant. The statistical associations between clinicopathological and immunohistochemical data were analysed by using contingency tables together with Fisher's exact test (2-sided) for all binary variables and the Chi-square test (2-sided) for all other nominal variables. In the subgroup of nonmuscle-invasive bladder cancer (NMIBC, pTa and pT1), progression-free survival (PFS) was evaluated. Stage-shifts (from pTa to pT1-4 or from pT1 to pT2-4) or the detection of distant metastasis was considered as progression. The Kaplan-Meier method was used for plotting the PFS curves. Significant differences between the curves were analysed by using the two-sided log-rank test. Associations between clinicopathological/immunohistochemical data and PFS were evaluated by univariable and multivariable Cox regression. All significant variables in the univariable analysis were used in the multivariable model.

## 3. Results

**Figure 1:**
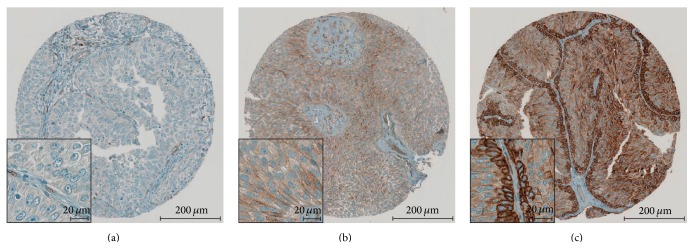
Immunohistochemical staining pattern of CD73. CD73 shows a distinct membranous immunoreactivity with pronounced intensity in the basal layer. Representative examples of score 0 (a), 1+ (b), and 2+ (c) are depicted.

All 174 (100%) tumor samples could be evaluated for CD73 immunoreactivity. CD73 showed a membranous immunoreactivity, which was much more pronounced in the basal layer than in the other tumor cells. Further, a faint and inhomogeneous cytoplasmatic staining was observed in most of the cases with positive membranous staining. In order to allow a distinct evaluation, only membranous CD73 immunoreactivity in the basal layer was assessed.  Figure 1 shows representative examples from our TMA for each staining score ranging from 0 to 2+.

A strong expression of CD73 (score 2+) was found in 46 of 174 patients (26.4%), whereas in 48 patients (27.6%) a weak immunoreactivity (score 1+) was detected. Finally, 80 patients (46%) showed no CD73 staining. The distribution of the dichotomized staining intensities of CD73 and Ki-67 are shown in Table 1. Additionally, we investigated 8 samples of normal urothelium (Supplementary Figures S1A–F in Supplementary Material available online at http://dx.doi.org/10.1155/2015/785461) of individuals without any history of urothelial dysplasia or carcinoma. We observed a weak and inhomogeneous expression of CD73 in the cytoplasm and cell membrane, which was more pronounced in the basal layer in 4 out of 6 cases (Supplementary Figures S1A–C, F).

Table 2 shows the associations between CD73 staining patterns and clinicopathological parameters (stage, grade, adjacent carcinoma in situ, multiplicity, growth pattern, and Ki-67) of the tumors. High CD73 expression is associated with lower stage (*p* = 0.006), lower grade (WHO 2004, *p* = 0.014), less adjacent carcinoma in situ (*p* = 0.007) and lower Ki-67 expression (*p* = 0.008). Contingency table analysis for the Ki-67 labelling index and the clinicopathological characteristics have been previously published [32] and showed a significant correlation (*p* < 0.05) with all clinicopathological characteristics except for tumor multiplicity.

**Figure 2:**
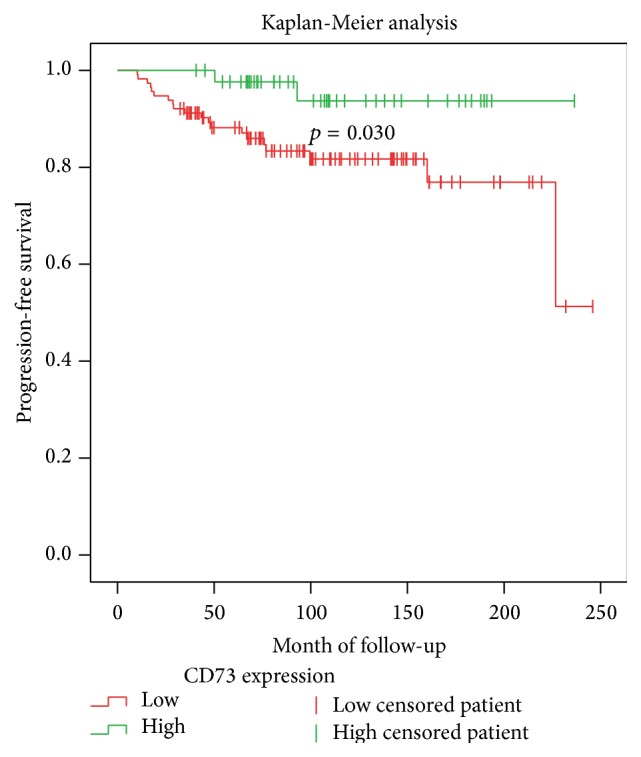
Kaplan-Meier analysis for progression-free survival comparing high and low CD73 expression. The log-rank test was used for the detection of statistical significance.

158 out of 174 patients (90.8%) underwent TUR for a primary pTa or pT1 urothelial carcinoma of the bladder. This subgroup was followed for a median of 110.7 months (range: 32.4–245.9 months). In total, 22 patients (13.9%) showed a stage-shift. The median time to progression was 45.2 months ranging from 10.2 to 226.7 months. Kaplan-Meier analysis shows that patients within the subgroup of low CD73 expression have a significantly shorter PFS compared to the subgroup of high CD73 expression (Figure 2). The corresponding log-rank test renders a *p* value of 0.030. Growth pattern (*p* < 0.001) and Ki-67 (*p* = 0.003) were also significantly associated with shorter PFS (Table 3).

In univariable Cox regression (Table 4), a high expression of CD73 reduces the rate of progression (hazard ratio, HR) by the factor of 0.228 (95% confidence interval [CI] ranging from 0.053 to 0.978; *p* = 0.047) compared to patients with a low expression of CD73. Of all other clinicopathologic parameters, growth pattern (HR = 7.634 [2.717–21.740]; *p* < 0.001) and Ki-67 (HR = 3.356 [1.429–7.874]; *p* = 0.006) were significantly associated with reduced PFS. We performed multivariable Cox regression analysis for all significant predictors in univariable analysis, where CD73 did not remain a significant predictor. Only growth pattern remained an independent predictor for shorter PFS (HR = 3.891 [1.245–12.195]; *p* = 0.019).

## 4. Discussion

This is the first study evaluating CD73 immunoreactivity in a large cohort of primary urothelial bladder carcinomas. We found that high CD73 expression is associated with lower stage, lower grade (WHO 2004), less adjacent carcinoma in situ and lower Ki-67 expression. In the subgroup of NMIBC (pTa and pT1), high CD73 immunoreactivity was associated with longer PFS in univariable Cox regression analysis but did not remain an independent predictor of longer PFS in multivariable Cox regression analysis.

Only a few studies have evaluated the role of CD73 in BC. Similar findings were reported by Wilson et al. who studied neoplastic transformed rodent bladders and detected a loss of CD73 after cancerous transformation [29]. A study using two human bladder cancer cell lines found a CD73 activity in the higher grade cell line of about five times as high as in the lower grade cell line [27]. Rockenbach et al. induced bladder cancer in mice and detected a higher expression of CD73 in the cancerous tissue [30]. One study evaluated CD73 enzyme activity in 36 human bladder cancer biopsies and 9 noncancerous bladder biopsies [28]. CD73 enzyme activity was comparable in BC and in normal urothelial tissue. However, contrary to our work, no follow-up data was reported on BC patients.

Several studies have investigated CD73 immunohistochemistry in other solid tumors. Interestingly, similar results have been reported for gynaecologic neoplasias: Oh et al. investigated 167 epithelial ovarian carcinomas and found associations between overexpression of CD73 and better prognosis, lower stage, and better differentiation [36]. Another study also assigning a favorable prognosis to elevated CD73 expression is the one of Supernat et al. that analysed 136 breast cancers (stages I–III) [37].

However, other investigations of CD73 in different solid tumors found contradictory results: CD73 has been reported as a disadvantageous prognostic or predictive factor, namely, in colorectal, gastric, gallbladder, prostate, and some forms of breast cancer [38–43].

Taken together, conflicting results have been published for the role of CD73 in solid tumors. Interestingly CD73 can be upregulated by several different mediators and conditions such as hypoxia [44, 45] and IFN-beta [46]. Hypoxia is a common characteristic of advanced cancers with poor progression while an increase in IFN-beta activates immunity against tumors [47]. Presence of hypoxia or IFN-beta in these tumors could partially explain the discrepancies. Furthermore, it is important to note that different tissues have very different enzymatic activity of CD73 [48]. This as well can potentially explain differences between different cancers.

In conclusion, our results are in contrast with the current proposed concepts from some authors, where overexpression of CD73 is considered as a disadvantageous factor in carcinogenesis [20, 25, 26]. In our study strong CD73 expression was associated with low-grade tumors, known for their good prognosis. Similar to our work, Oh and Rackley et al. could also detect firstly an inverse relationship between grading and expression of CD73 in epithelial ovarian carcinomas and prostate cancers, respectively, and secondly a direct relationship between expression of CD73 and advantageous prognosis [36, 49]. As in our work, strong CD73 expression was also proposed as a potential marker of good prognosis in breast cancer [37]. Very recently, a molecular biologic link between BC and breast cancer has been found, where a study identified two intrinsic, molecular subsets of high-grade BC, which have similar characteristics of subtypes of breast cancer [50]. The results of this work suggest that molecular characteristics of BC reflect many aspects of breast cancer. Since CD73 promotes the hydrolysis of AMP into adenosine and phosphate [20, 21], the results of our study could also be explained by the proapoptotic effect of adenosine postulated by several articles [51–53].

In our study, high CD73 expression is associated with lower stage and grade and therefore with low risk disease. However, we note that low CD73 expression is also present in a distinct amount of patients with pTa tumors (*n* = 57, 33%; see Table 2). According to our results presented in Table 2, it seems that strong expression of CD73 has good specificity (0.85) but low sensitivity (0.63) in the prediction of low risk (pTa) tumors.

There are limitations to our study. First, this is a retrospective, single-institution study. Second, information about adjuvant instillation therapy could not be evaluated. As tumor recurrence and progression is known to vary upon instillation therapy, it is an important missing aspect of this study. Also, the investigated study population has been collected over a long time period between 1990 and 2006 where clinical practice with respect to intravesical therapy has changed. Thus, this may have additionally influenced our study results. However, all patients had primary (nonrecurrent) tumors and a long follow-up period, which makes this study population nevertheless meaningful to study. Third, the low rate of MIBC patients has to be mentioned. Hence, no conclusion can be drawn for this group of tumors. Prospective studies with a higher proportion of MIBC are necessary to further evaluate the role of CD73 as a biomarker in BC. Furthermore—beside validation studies for the function of CD73 as a biomarker in BC—more research investigating the role of CD73 as a possible therapeutic target is warranted.

## 5. Conclusion

High expression of CD73 is observed in BC patients with favorable pathological features. In NMIBC, high CD73 expression was additionally associated with good prognosis. Besides clinical and pathological parameters, detection of strong CD73 expression in BC may help to stratify patients into a low risk group. Further studies are warranted to confirm this hypothesis.

## Supplementary Material

Supplemental Figure 1: Normal urothelium (A-F) including von Brunn's nests (E-F) showing a weak and inhomogeneous, partly cytoplasmic and partly membranous immunoreactivity of CD73. The expression of CD73 is slightly accentuated in the basal layer in 4 out of 6 cases (A-C, F).

## Figures and Tables

**Table 1:** Patient and tumor characteristics and results of immunohistochemical analyses.

Variable	Categorization	*n* analyzable^a^	%
Total (*n* = 174)^a^			
Clinicopathologic data			
Age at diagnosis (median, range): 69.5 years (32–92)	<70 years	87	50.0
	≥70 years	87	50.0
Sex	Female	43	24.7
	Male	131	75.3
Tumor stage (WHO 1973^b^)	pTa	90	51.7
	pT1	68	39.1
	pT2	13	7.5
	pT3	2	1.1
	pT4	1	0.6
Histologic grade (WHO 1973^b^)	G1	44	25.3
	G2	87	50.0
	G3	43	24.7
Histologic grade (WHO 2004^c^)	Low grade	101	58.0
	High grade	73	42.0
Adjacent carcinoma in situ	No	158	90.8
	Yes	16	9.2
Multiplicity	Solitary	124	71.3
	Multifocal	50	28.7
Growth pattern	Papillary	159	91.4
	Solid	15	8.6

Immunohistochemistry (IHC)			
CD73	Score 0	80	46.0
	Score 1+	48	27.6
	Score 2+	46	26.4
Ki-67 labelling index	≤10%	108	62.1
	>10%	66	37.9

^a^All patients.

^b^Staging and grading according to the 1973 WHO classification system.

^c^Staging and grading according to the 2004 WHO classification system.

**Table 2:** Comparison of the immunohistochemical markers with pathologic characteristics (*n* = 174).

Variable	Categorization	CD73 expression		
		Score 0 or 1+	Score 2+	*p*
Tumor stage (WHO 1973)^a^	pTa	57	33	**0.006**
	pT1	57	11	
	pT2	12	1	
	pT3	2	0	
	pT4	0	1	
Histologic grade (WHO 1973)^a^	G1	31	13	0.100
	G2	60	27	
	G3	37	6	
Histologic grade (WHO 2004)^b^	Low grade	67	34	**0.014**
	High grade	61	12	
Adjacent carcinoma in situ^b^	No	112	46	**0.007**
	Yes	16	0	
Multiplicity^b^	Solitary	87	37	0.130
	Multifocal	41	9	
Growth pattern^b^	Papillary	115	44	0.359
	Solid	13	2	

Immunohistochemistry				
Ki-67 labelling index	≤10%	72	36	**0.008**
	>10%	56	10	

^a^Chi-square Pearson (2-sided); bold face representing *p* values <0.05.

^b^Fisher's exact test (2-sided); bold face representing *p* values <0.05.

**Table 3:** Analysis of factors for tumor progression.

Variable	Categorization	Tumor progression (TP)		
		*n* ^a^	Events	*p* ^b^
Pathologic data				
Tumor stage (WHO 1973^c^)	pTa	90	10	0.360
	pT1	68	12	
Histologic grade (WHO 1973^c^)	G1	44	3	0.085
	G2	86	12	
	G3	28	7	
Histologic grade (WHO 2004^d^)	Low grade	99	10	0.083
	High grade	59	12	
Adjacent carcinoma in situ	No	146	20	0.545
	Yes	12	2	
Multifocality	Unifocal tumor	115	15	0.465
	Multifocal tumor	43	7	
Growth pattern	Papillary	151	17	**<0.0001**
	Solid	7	5	

Immunohistochemistry				
CD73	Score 0 or 1+	114	20	**0.030**
	Score 2+	44	2	
Ki-67 labelling index	≤10%	106	9	**0.003**
	>10%	52	13	

^a^Only primary pTa and pT1 tumors are included.

^b^Log-rank test (2-sided); bold face representing *p* values <0.05.

^c^Staging and grading according to the 1973 WHO classification system.

^d^Staging and grading according to the 2004 WHO classification system.

**Table 4:** Regression analysis.

Variable (categorization)	Univariable analysis				Multivariable analysis			
	HR	95% CI		*p* value	HR	95% CI		*p* value
Tumor stage (pTa versus pT1)	1.481	0.635	3.454	0.363				
Histologic grade (WHO 2004^a^)	2.070	0.894	4.808	0.090				
Adjacent carcinoma in situ	1.563	0.363	6.711	0.549				
Multifocality	1.401	0.565	3.472	0.467				
Growth pattern (papillary versus solid)	7.634	2.717	21.740	**<0.001**	3.891	1.245	12.195	**0.019**
CD73	0.228	0.053	0.978	**0.047**	0.319	0.072	1.408	0.132
Ki-67	3.356	1.429	7.874	**0.006**	2.222	0.873	5.650	0.094

^a^Staging and grading according to the 2004 WHO classification system.
