# Supplementary figures and images for: CD73 Predicts Favorable Prognosis in Patients with Nonmuscle-Invasive Urothelial Bladder Cancer
Source: Dis Markers. 2015 Oct 12;2015:785461. doi: 10.1155/2015/785461 (PMC4620269; doi:10.1155/2015/785461)

**A**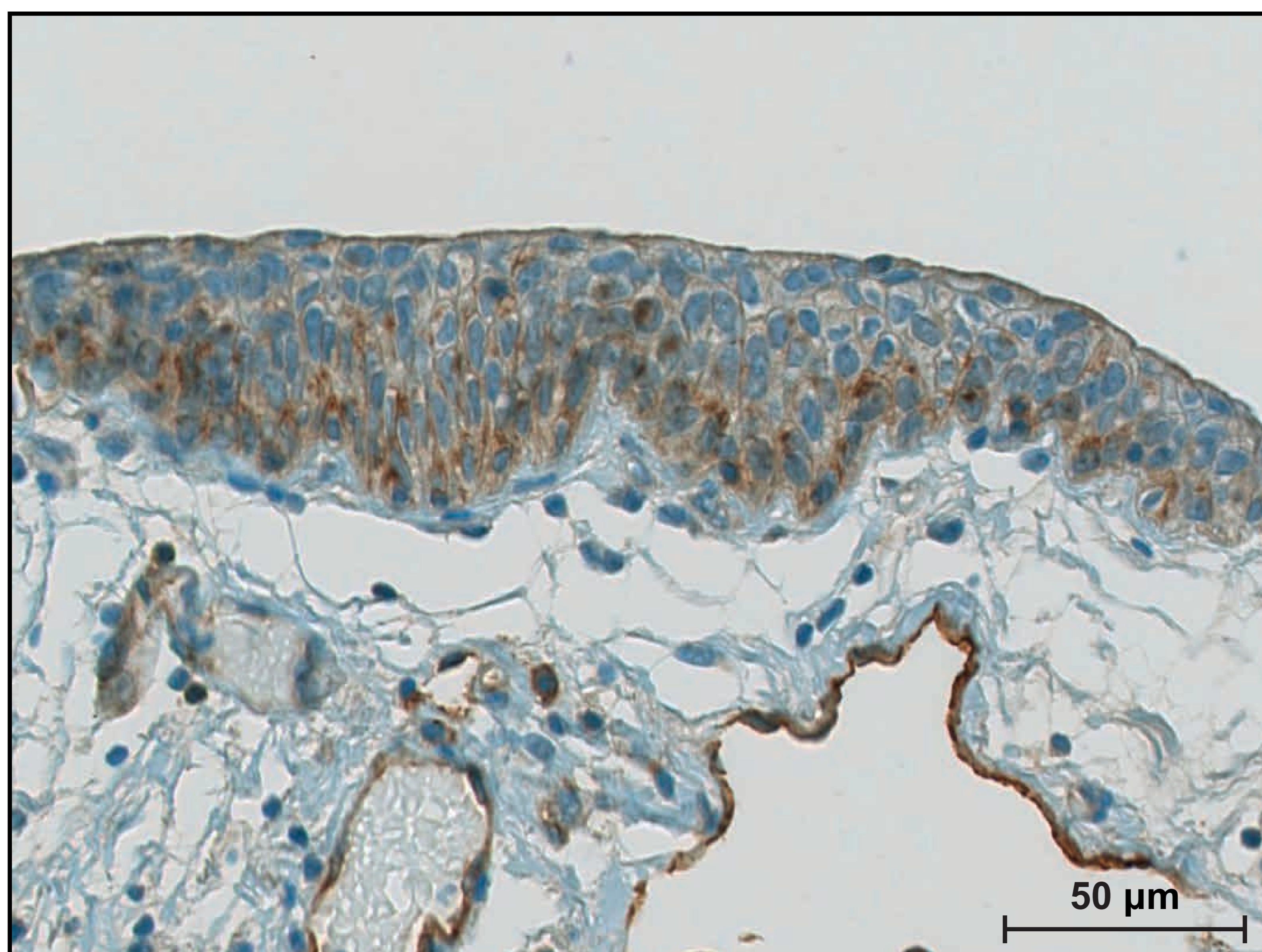**B**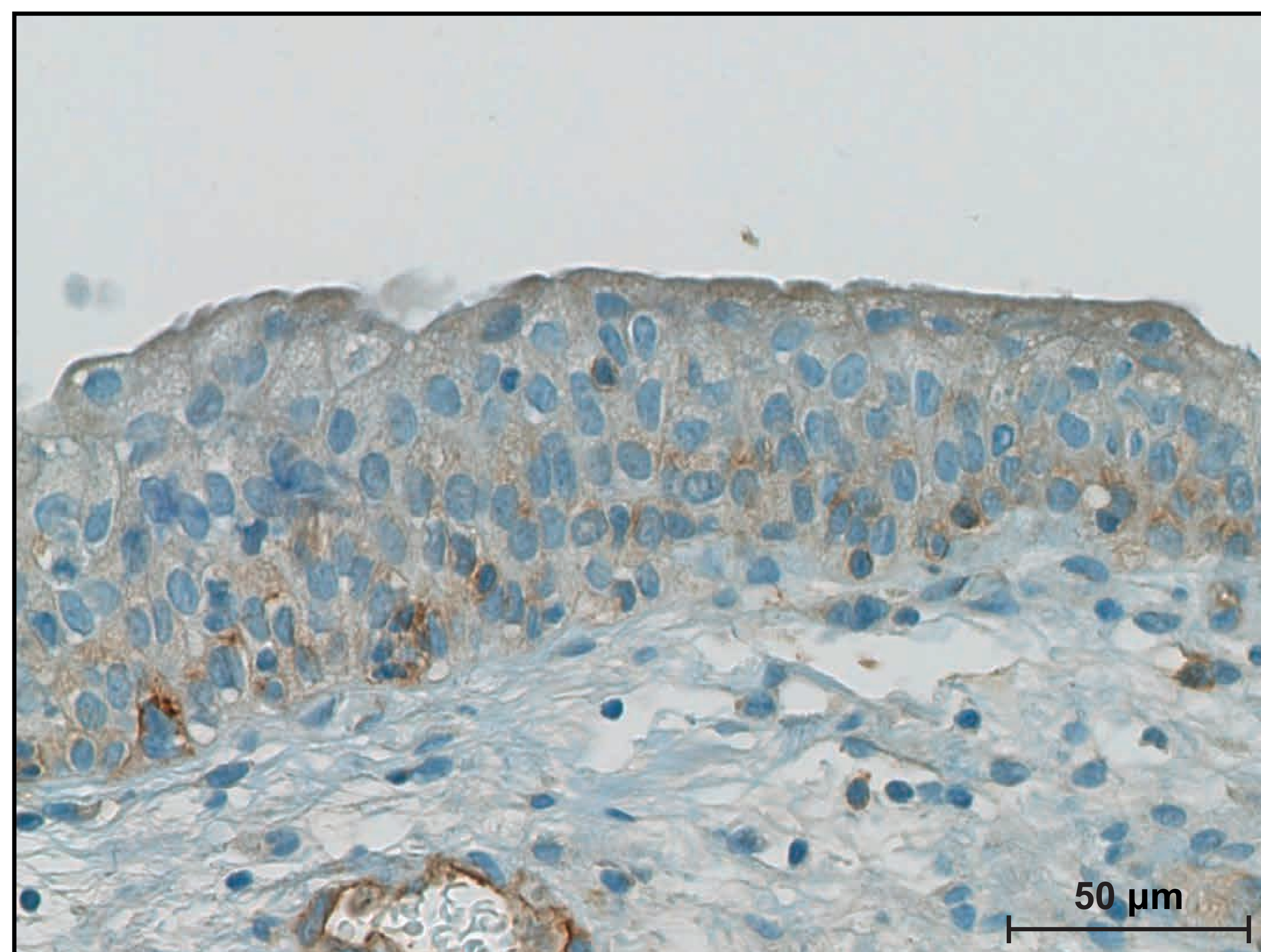**C**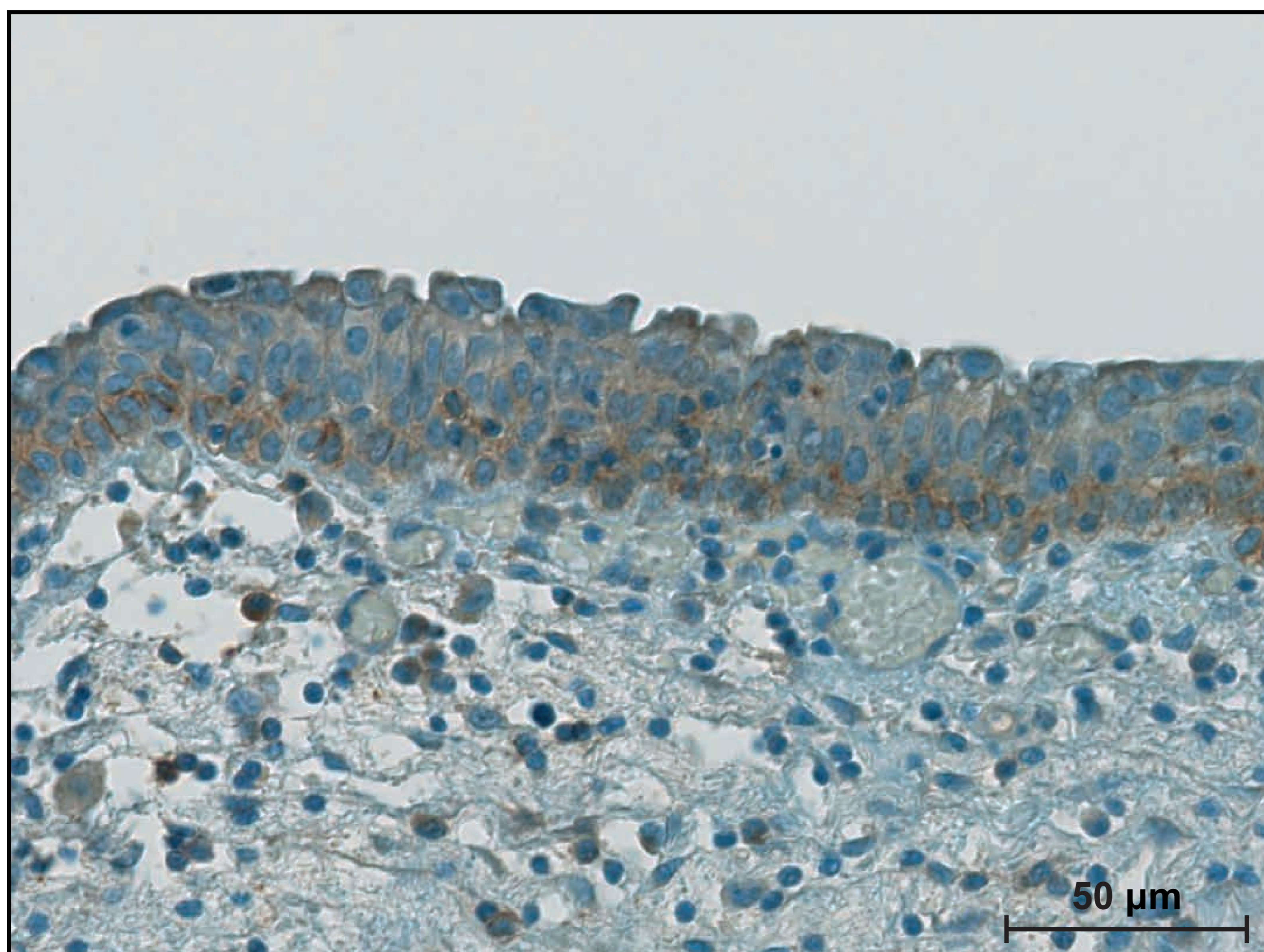**D**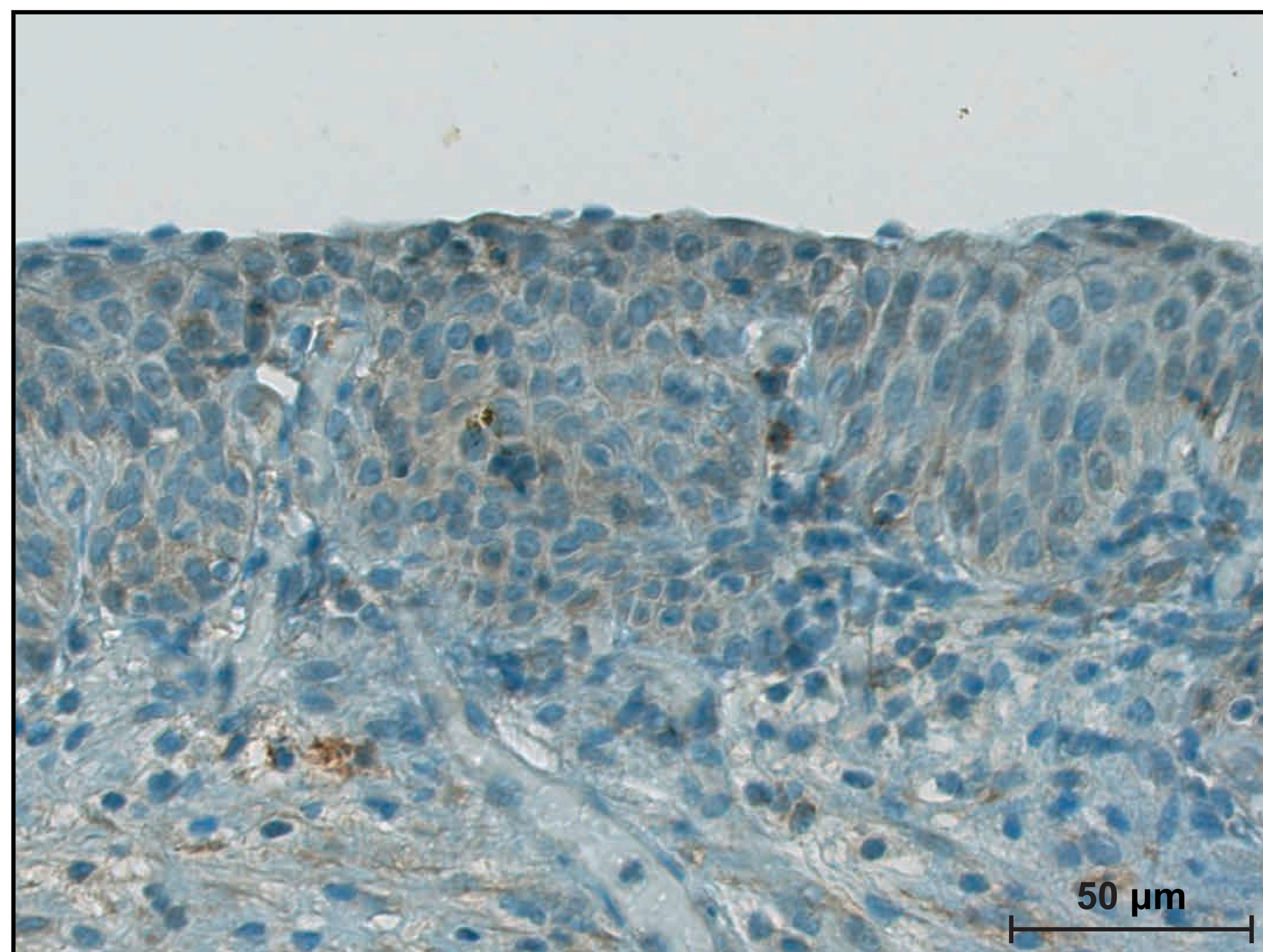**E**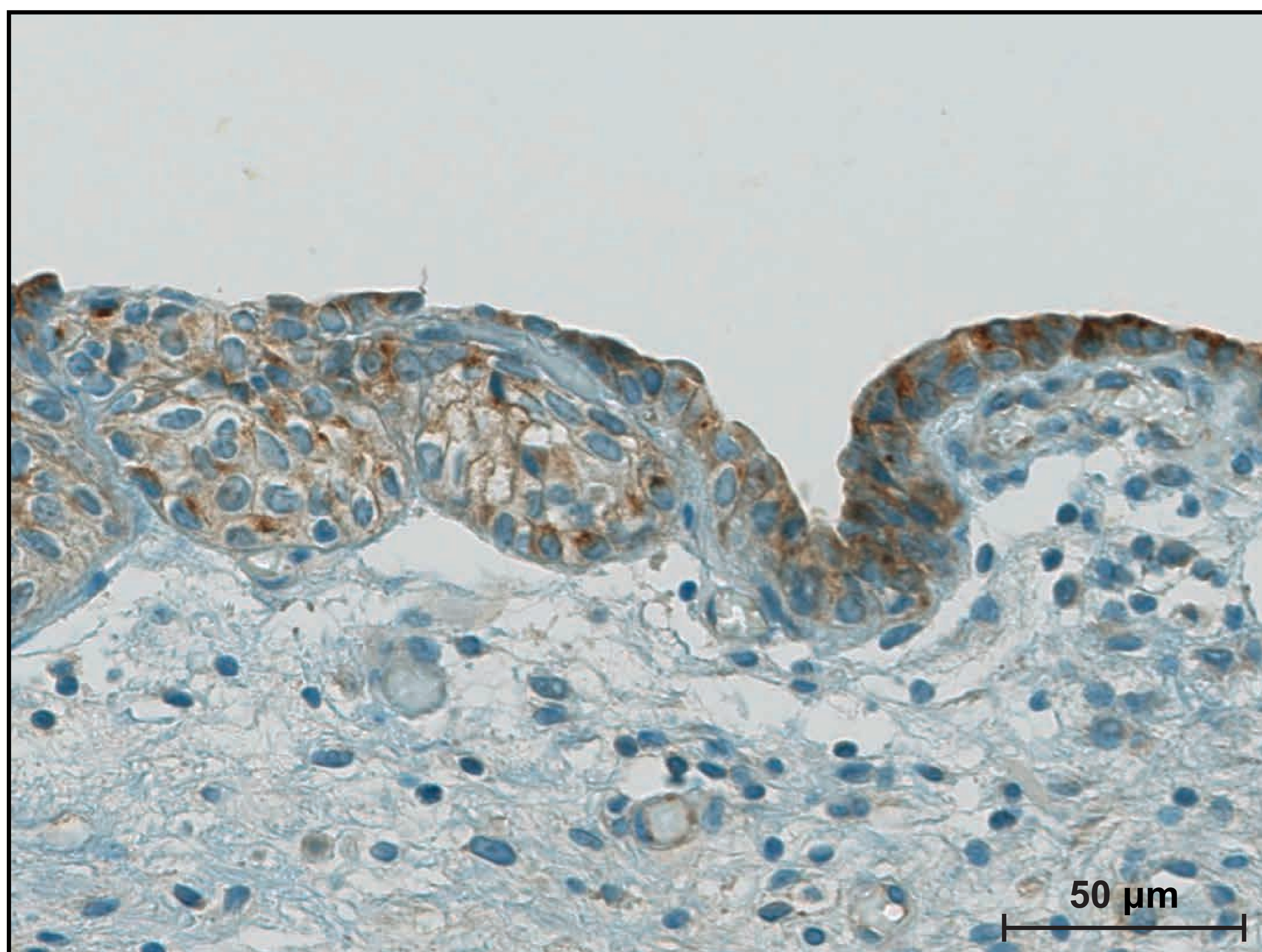**F**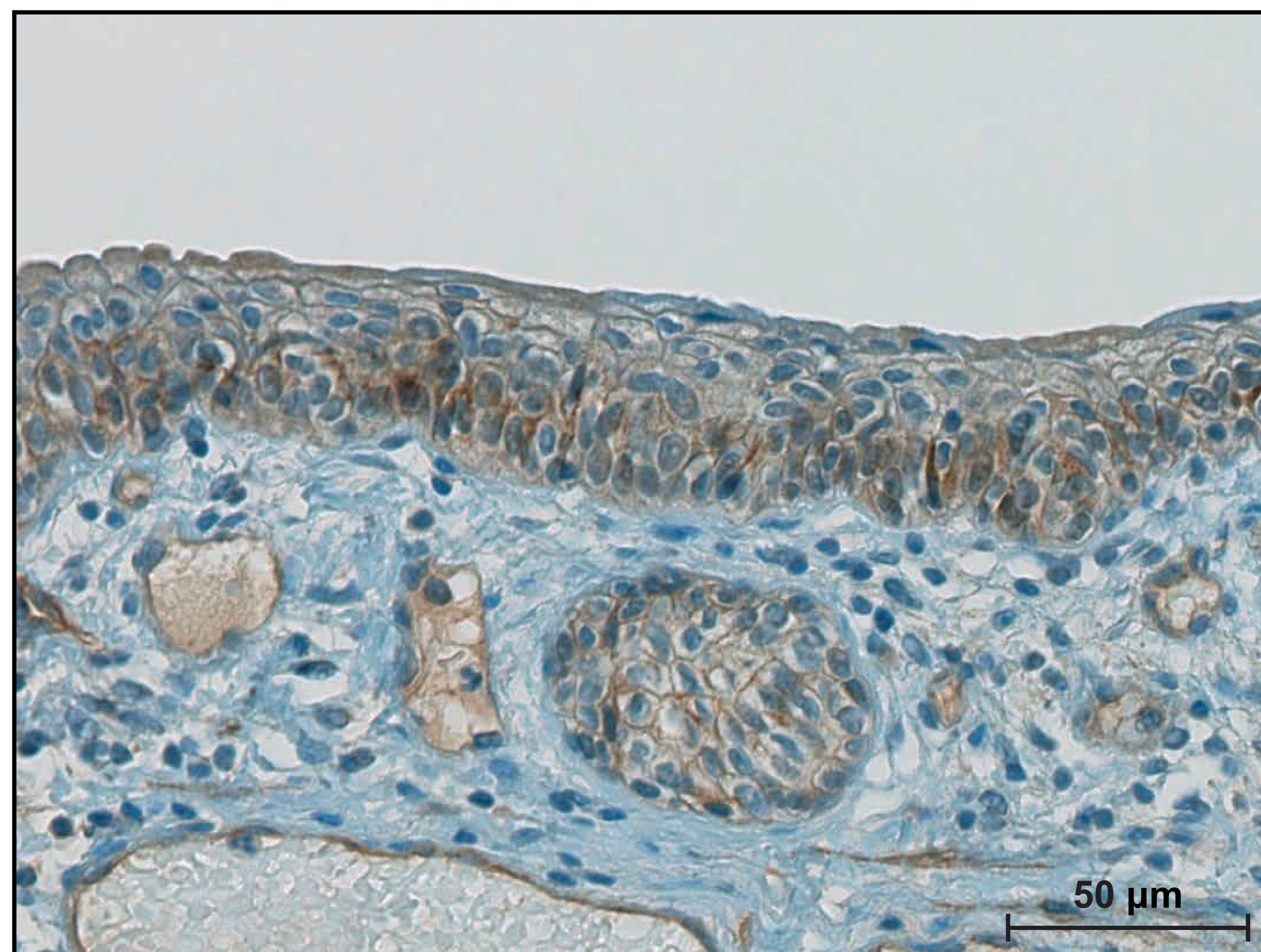

Supplement: Supplementary file 1 — Supplemental Figure 1: Normal urothelium (A-F) including von Brunn's nests (E-F) showing a weak and inhomogeneous, partly cytoplasmic and partly membranous immunoreactivity of CD73. The expression of CD73 is slightly accentuated in the basal layer in 4 out of 6 cases (A-C, F). [file 785461.f1.pdf]
